# *Wolbachia* in Antarctic terrestrial invertebrates: Absent or undiscovered?

**DOI:** 10.1111/1758-2229.70040

**Published:** 2024-11-13

**Authors:** Svitlana Serga, Pavlo A. Kovalenko, Oleksandr M. Maistrenko, Gwenaëlle Deconninck, Oleksandra Shevchenko, Nataliia Iakovenko, Yurii Protsenko, Andrij Susulovsky, Łukasz Kaczmarek, Mariia Pavlovska, Peter Convey, Iryna Kozeretska

**Affiliations:** ^1^ CBGP, Univ Montpellier, CIRAD, INRAE, IRD Institut Agro Montpellier Montpellier France; ^2^ National Antarctic Scientific Center of Ukraine Kyiv Ukraine; ^3^ State Institution Institute for Evolutionary Ecology National Academy of Sciences of Ukraine Kyiv Ukraine; ^4^ European Molecular Biology Laboratory Structural and Computational Biology Unit Heidelberg Germany; ^5^ Royal Netherlands Institute for Sea Research, 't Horntje (Texel) Den Hoorn Netherlands; ^6^ UMR CNRS 7261 Institut de Recherche sur la Biologie de l'Insecte Université de Tours, Parc Grandmont Tours France; ^7^ Institute for Problems of Cryobiology and Cryomedicine National Academy of Sciences of Ukraine Kharkiv Ukraine; ^8^ I.I. Schmalhausen Institute of Zoology National Academy of Sciences of Ukraine Kyiv Ukraine; ^9^ Czech University of Life Sciences Prague Faculty of Forestry and Wood Sciences Suchdol Czech Republic; ^10^ Institute of Animal Physiology and Genetics AS ČR Laboratory of Nonmendelian Evolution Libechov Czech Republic; ^11^ Taras Shevchenko National University of Kyiv Kyiv Ukraine; ^12^ State Museum of Natural History National Academy of Sciences of Ukraine Lviv Ukraine; ^13^ Department of Animal Taxonomy and Ecology, Faculty of Biology Adam Mickiewicz University in Poznań Poznań Poland; ^14^ British Antarctic Survey, NERC, High Cross Cambridge UK; ^15^ Department of Zoology University of Johannesburg Auckland Park South Africa; ^16^ Biodiversity of Antarctic and Sub‐Antarctic Ecosystems (BASE) Santiago Chile

## Abstract

Interactions between a host organism and its associated microbiota, including symbiotic bacteria, play a crucial role in host adaptation to changing environmental conditions. Antarctica provides a unique environment for the establishment and maintenance of symbiotic relationships. One of the most extensively studied symbiotic bacteria in invertebrates is *Wolbachia pipientis*, which is associated with a wide variety of invertebrates. *Wolbachia* is known for manipulating host reproduction and having obligate or facultative mutualistic relationships with various hosts. However, there is a lack of clear understanding of the prevalence of *Wolbachia* in terrestrial invertebrates in Antarctica. We present the outcomes of a literature search for information on the occurrence of *Wolbachia* in each of the major taxonomic groups of terrestrial invertebrates (Acari, Collembola, Diptera, Rotifera, Nematoda, Tardigrada). We also performed profiling of prokaryotes based on three marker genes and Kraken2 in available whole genome sequence data obtained from Antarctic invertebrate samples. We found no reports or molecular evidence of *Wolbachia* in these invertebrate groups in Antarctica. We discuss possible reasons underlying this apparent absence and suggest opportunities for more targeted future research to confirm bacteria's presence or absence.

## Graphical abstract

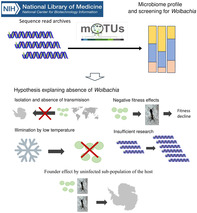

## INTRODUCTION

The key role that microbiota plays in their hosts' physiology and adaptation is increasingly recognized (McFall‐Ngai et al., 2013). These roles are mediated by the specific microbial community composition and interactions therein. For example, the gut microbial community plays a central role in host nutrition and immunity (Gilbert et al., 2012). Relationships with symbiotic bacteria, from obligate symbiosis to facultative interactions under specific environmental conditions, are linked with a wide range of effects on the host organism, influencing its fitness (Moran, 2007). Symbiotic bacteria have the potential to improve the ability of the host organism to adapt to changing environments or, under certain conditions, can negatively influence their survival. Furthermore, environmental factors themselves can impact symbiotic relationships. While some microorganisms are sensitive to very small environmental changes, others are resilient to a wide range of different conditions (Bénard et al., 2020; Putnam et al., 2017).

*Wolbachia pipientis* Hertig and Wolbach, 1924, a member of the bacterial order Rickettsiales (Alphaproteobacteria), is one of the most widespread symbiotic bacteria present across invertebrate phyla and is reported to infect 48%–57% of all terrestrial arthropod species (Weinert et al., 2015). Generally referred to simply as *Wolbachia*, it is a key example of a symbiont with wide range of phenotypic effects on different host species, spanning mutualistic, symbiotic and parasitic relationships (reviewed by Kaur et al., 2021 and Mioduchowska et al., 2023). The bacteria are generally transmitted vertically via the maternal germ line (Werren et al., 2008), although alternative routes of transmission, including horizontal transfer between host species, hybrid introgression and codivergence, have been repeatedly noted (Baldo et al., 2008; Raychoudhury et al., 2009).

The species *W. pipientis* is currently divided into 20 supergroups, named A–F, H–Q and S–V, based on sequence divergence and phylogenetic relationships (Mioduchowska et al., 2023), although this classification remains debated and continues to develop (Lindsey et al., 2016; Mahmood et al., 2023; Ramírez‐Puebla et al., 2015). Most of these supergroups are restricted to arthropods. Exceptions to this include C, D, J and L, whose members infect nematodes, F, which has been reported to infect both nematodes and arthropods (Lefoulon et al., 2016; Lo et al., 2002; Mioduchowska et al., 2023), A, which infects arthropods, tardigrades and molluscs, and V, which infects molluscs and arthropods (Mioduchowska et al., 2023). The divergent supergroup E (Vandekerckhove et al., 1999) is predominantly found specifically in asexually reproducing Collembola (springtails), although other supergroups are also found in sexually reproducing springtails (Rodrigues et al., 2023; Timmermans et al., 2023). Each supergroup contains a number of distinct *Wolbachia* strains, with most being separable by analysis of the sequence of the *Wolbachia surface protein* (*wsp*) gene or by multilocus sequence typing (Baldo et al., 2006; Braig et al., 1998).

In different host species, *Wolbachia* can act as a reproductive parasite or a facultative or obligate symbiont (Kaur et al., 2021; Zug & Hammerstein, 2015), frequently having both beneficial and detrimental effects concurrently (Zug & Hammerstein, 2015). The evolutionary success of *Wolbachia* in its various arthropod host species primarily relies on its mechanisms of influencing host reproduction, including cytoplasmic incompatibility (CI), male killing (MK), feminization of genetic males and induction of parthenogenesis (O'Neill et al., 1997). Both the nature and intensity of reproductive manipulation depend on the specific *Wolbachia* strain involved and the host's genetic background (Braig et al., 1994; Veneti et al., 2012). Obligate symbiotic representatives of *Wolbachia* are present in many phylogenetically distinct taxa. For instance, in filarial nematodes *Wolbachia* acts as a nutritional symbiont (Taylor et al., 2013), while it plays a vital role in the normal development of eggs in the parasitic wasp, *Asobara tabida* Nees, 1834 (Dedeine et al., 2001). Beneficial effects of *Wolbachia* infection on fitness and stress response traits have also been reported, such as resistance to infection by viruses and *Plasmodium* (Bourtzis et al., 2014; Hedges et al., 2008; Pimentel et al., 2021; Teixeira et al., 2008), increased fecundity (Ju et al., 2020; Serga et al., 2014), the promotion of stress resistance (Lau et al., 2020), nutrient provisioning (Ju et al., 2020; Newton & Rice, 2020) and improved tolerance of diet shifts (Deconninck et al., 2024). In other instances, *Wolbachia* infection leads to negative effects on host fitness, such as decreased lifespan (Martinez et al., 2015; Min & Benzer, 1997) or reduced cold resistance (Serga et al., 2021). The direction and strength of such effects can be very variable, depending on both host genetic background and environmental conditions (Serga et al., 2021; Strunov et al., 2022).

Environmental temperature can affect *Wolbachia* prevalence in different ways, such as through direct effects on the bacteria itself (reviewed by Shropshire et al., 2020), effects on host distribution (Lau et al., 2020) or symbiont transmission efficiency (Hague et al., 2022). Studies of climate influence on *Wolbachia* prevalence at broad geographical and taxonomic scales have identified complex patterns, depending on the host group and climatic zone (Charlesworth et al., 2019). In chelicerates, infection rates are consistent across different geographical regions and host groups (Charlesworth et al., 2019). In insects, infection rates broadly increase with temperature, although this relationship is limited to the temperate zone and lower infection rates were generally found in tropical regions (Charlesworth et al., 2019). In contrast to this general pattern, analyses of *Wolbachia* infection rates in the well‐sampled species, *Drosophila melanogaster* Meigen, 1830, suggested that they were higher in the tropics and lower in temperate regions (Kriesner et al., 2016), as well as being sensitive to different combinations of climatic factors in different continents (Gora et al., 2020).

There is considerable evidence that temperature influences *Wolbachia* titers in the host organism (Chrostek et al., 2021; Clancy & Hoffmann, 1998; Foo et al., 2019; Hague et al., 2020), but relationships between bacterial titers and effects on the host organism are very variable and also depend on the host species (reviewed by Shropshire et al., 2020). Temperature has a significant impact on maternal transmission success. For example, transmission is lower in *D. melanogaster* at 20°C than at 25 or 28°C (Hague et al., 2022). An indirect influence of temperature on the *Wolbachia*‐host association is also possible, as the bacteria affect host fitness under certain temperature regimes which, in turn, can affect the spread and persistence of infection in the natural environment (Lau et al., 2020; Serga et al., 2021). Therefore, *Wolbachia* appears to be a temperature sensitive bacterium.

Under stress, cells of all organisms activate a range of stress responses. After being exposed to cold stress, *Wolbachia*‐infected *D. melanogaster* showed higher expression of CRP genes (*Heat‐shock‐protein‐70Aa* [*Hsp70Aa*] and *Autophagy‐related gene‐1* [*Atg1*]) in comparison with *Wolbachia*‐free flies (Camerota et al., 2015). *Wolbachia*‐infected *Aedes aegypti* Linnaeus, 1762 performed better at low temperatures than did uninfected mosquitoes (Lau et al., 2020). Serga et al. (2021) reported an effect of *Wolbachia* infection on chill coma recovery time in only one of seven tested genotypes of *D. melanogaster* in 21‐day old flies, with infected flies recovering more slowly from chill coma. Such studies suggest that the impact of *Wolbachia* infection on cold tolerance may apply only to specific genotypes.

## THE ANTARCTIC ENVIRONMENT AND ITS TERRESTRIAL BIODIVERSITY

The extreme conditions that are typical for the Antarctic region are a paradigmatic example of environmental extremes on Earth, and have long been recognized and used as a tool to help understand mechanisms of adaptation to environmental stress. Antarctic terrestrial biodiversity is low, consisting almost entirely of invertebrates, lower plants, lichens and microbiota (Convey et al., 2014; Convey & Biersma, 2024). Microbial diversity is particularly poorly known and understood in Antarctica (Cavicchioli, 2015; Chown et al., 2015), including both free‐living forms (e.g., the soil microbiome) and those associated with other organisms (e.g., the invertebrate microbiome or plant rhizosphere), with baseline survey data and up‐to‐date taxonomic treatments still lacking. Terrestrial invertebrates in Antarctica are represented by arthropods, nematodes, rotifers and tardigrades, a large majority of which appear to be endemic to different regions within the continent and its associated islands (Convey et al., 2020). According to RAS (2024) two classes of terrestrial arthropod are present, Arachnida (subclass Acari) and Hexapoda (subclasses Collembola and Insecta), each represented by five orders. Of the five orders of Hexapoda, two are free‐living (Collembola and Diptera), two are permanently parasitic (Anoplura and Mallophaga) and one (Siphonaptera) spends part of its life cycle in seasonally abandoned nests of birds (Convey, 2017; Gressitt, 1965; Obbels et al., 2016).

To date, no reports are available of the occurrence of *Wolbachia* in Antarctic invertebrates, and it is unclear whether this represents the true absence of the bacteria or is a consequence of a lack of research. It might be hypothesized that Antarctica provides a particularly suitable environment for the establishment and maintenance (or possibly absence) of symbiotic relationships due to its multiple extreme conditions (e.g., low temperatures; significant ozone hole‐related ultraviolet radiation variation: from high between September and December to low for the rest of the year; desiccation [especially in the continental Antarctic]; low nutrients [Convey & Peck, 2019; Convey et al., 2014]), consistently low terrestrial species diversity (lower species diversity giving fewer opportunities for horizontal transmission) and geographical isolation from other continents and landmasses (i.e., the establishment of new species is extremely infrequent). It is known that *Wolbachia* titers are nutrient‐sensitive (Padde et al., 2023; Serbus et al., 2015) and temperature‐sensitive (Chrostek et al., 2021; Clancy & Hoffmann, 1998; Foo et al., 2019; Hague et al., 2020), and that this bacterium reduces host resistance to desiccation in mosquitoes (Allman et al., 2020; Farnesi et al., 2019). However, no studies have investigated the effects of ultraviolet radiation on *Wolbachia* spread and its influence on host species. How these factors, individually or in combination, may affect host‐symbiont interactions in Antarctica is unknown, while there remains a lack of systematic research targeting the presence of endosymbionts in Antarctic invertebrate hosts.

## EXPERIMENTAL PROCEDURES

To address this lack of knowledge, we searched the published literature for PCR‐ or sequencing‐based studies screening for *Wolbachia* in invertebrate species present in Antarctica (Table S1). We first searched using a combination of the keywords “*Wolbachia*” and “Antarctica” and “Rickettsiales” and “Antarctica” in the PubMed and Web of Science Databases. Second, we used “*Wolbachia*” and each invertebrate group name (e.g., “Acari”, “Collembola”, etc.) and, finally, we searched the databases using the keywords “*Wolbachia*” and individual invertebrate taxon names (e.g., “Oribatida”). The literature search was completed on 24 January, 2024. Additionally, we searched the PubMed and Web of Science Databases with the combination of keywords “*Wolbachia*” and “Arctic” and “Rickettsiales” and “Arctic”, as the Arctic represents the most relevant region globally for comparison with similar extreme environmental conditions. From each study identified, we extracted information on host species taxonomy, method of *Wolbachia* identification, numbers of tested individuals and SRAs (sequence read archives), life stage and sex, sample origin (including GPS coordinates) and year of collection. We also consulted the largest database currently available of *Wolbachia* records in terrestrial arthropods from different geographical regions globally (Weinert et al., 2015).

We conducted an extensive search of recent soil microbiome sequencing data and found no studies mentioning *Wolbachia* presence in the Antarctic soil samples. Moreover, the PubMed search for the order *Rickettsiales* in Antarctica gave no relevant results. This either means that *Wolbachia* is absent in the Antarctic environment, or that the scarcity of this type of data available to date does not allow for detection of this genus.

We also note that, even if *Wolbachia* were to be assigned in soil eDNA studies, such reports should still be treated with caution. While the eDNA approach is sensitive, independent and cost‐efficient, it still suffers from false positives, whilst assignments are based on an assessment of sequence similarity, inherently relying on completeness and accuracy of sequence databases, and not confirming the presence or viability of an actual living organism or propagules. The putative reasons for false positive results include but are not limited to insufficient assay specificity, contamination and persistence of DNA in the environment after its introduction from an exogenous source (Evans et al., 2017; Rishan et al., 2023).

We also searched for evidence of *Wolbachia* presence in Antarctic invertebrates by examining whole‐genome sequencing data for bacterial identification (Pascar & Chandler, 2018; Scholz et al., 2020; Vancaester & Blaxter, 2023). We downloaded publicly available SRAs (whole genome sequencing data obtained using Illumina technology) obtained from invertebrates known to occur in Antarctica by searching for them by species name, although the majority of the specific samples reported were obtained from non‐Antarctic parts of their distributions (5 SRA libraries from Antarctic region and 150 SRA libraries from non‐Antarctic areas; Table S2). We performed profiling of the prokaryote sequence diversity present with the mOTU3 profiler (https://github.com/motu-tool/mOTUs) using three marker genes (Milanese et al., 2019; Ruscheweyh et al., 2022). To further explore the read archive data and confirm findings with the mOTU3 profiler, we investigated classification profiles provided by the NCBI sequence read archive classifier (https://www.ncbi.nlm.nih.gov/sra/) and Kraken2 (Wood et al., 2019) (in this test, we analysed a total of 189 libraries, including 34 additional SRA libraries of *Belgica antarctica* Jacobs, 1900 from Antarctica, which were previously assessed using the mOTU3 profiler by Maistrenko et al. (2023); Table S2). We used the “Standard” version 6/5/2024 of the Kraken2 database downloadable from https://benlangmead.github.io/aws-indexes/k2 which includes Refseq archaea, bacteria, viral, plasmid, and human genomes. Evaluation of the presence of *Wolbachia* for this article is qualitative. For the mOTU3 profiler if abundance was >0% based on marker genes we consider *Wolbachia* potentially present; for the NCBI‐classifier and Kraken2 if *Wolbachia* genome coverage was >0.01% and >0% respectively we considered *Wolbachia* potentially present. For relative abundance, we used estimates from the mOTU3 pipeline abundance report. The code is available on GitHub (https://github.com/omaistrenko/WolbachiaInAntarcticTerrestrialInvertebrates).

## RESULTS

The literature search identified that *Wolbachia* infection had been tested for in 23 samples obtained from Antarctica, representing at least seven species of terrestrial invertebrates (in total, >1306 individuals) (Table 1). Different methods were used in these studies, including PCR screening (eight samples), 16S rRNA sequence analyses (11 samples) and whole‐genome sequencing (four samples) (Table S1). However, none of these studies reported or inferred *Wolbachia* infection in any of the Antarctic invertebrates examined.

**TABLE 1:** Terrestrial invertebrates screened for *Wolbachia* presence in samples obtained from Antarctica.

Taxonomic group	Literature search			Bioinformatics search		
	Number of tested species	Number of tested samples^a^	Number of tested individuals^b^	Number of tested species	Number of tested SRA	Number of tested individuals^c^
Acari	1	3	96	0	0	0
Collembola	1	1	1–3	0	0	0
Diptera	1	12	>249	1	4	20
Nematoda	2	2	264	1	1	5000
Tardigrada	At least 2 determined	5	696	0	0	0
Total	At least 7 determined	23	>1306	2	5	5020

^a^ If individuals of one species, one life stage and one sex were collected from one locality at the same time, it is considered one sample.

^b^ If among the studied samples there was at least one where the exact number of individuals was not known, then the sign > is used for the total number of individuals.

^c^ The number of individuals used for the DNA extractions to generate SRA libraries was found in the original articles describing protocols for sample preparation and sequencing (Kim et al., 2017; Xue et al., 2021).

**FIGURE 1:**
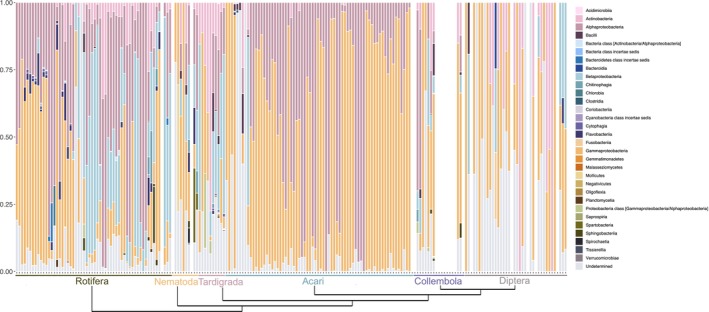
Relative abundance profiles of microbiome associated with invertebrate species which are present in Antarctic biomes.

We downloaded 155 SRA libraries from publicly available databases, which were obtained from terrestrial invertebrates known to occur in Antarctica. However, only five libraries were generated from samples collected in the Antarctic region itself (Table S2). No *Wolbachia* sequence assignments were reported from any of the samples analysed (Table 1, Figure 1, Table S3) using the mOTU3 profiler. The NCBI SRA classifier reports the *Collembola* species *Folsomia candida* V.Willem, 1902 (6/18) to contain *Wolbachia*, however all specimens were collected outside of Antarctica (see Table S2). These results are in agreement with the data mining for endosymbionts across publicly available sequencing data reported by Medina et al. (2023) and Scholz et al. (2020). According to the NCBI classifier, several other specimens contain hits to *Wolbachia* with low horizontal coverage (see Table S2): *Anisakis simplex* (Rudolphi, 1809) Dujardin, 1845 (1/4); *Adineta vaga* (Davis, 1873) (1/28); *B. antarctica* (1/34); *Brachionus calyciflorus* Pallas, 1766 (2/10); *Halotydeus destructor* (Tucker, 1925) (3/61; although this particular species is not reported from Antarctica, the presence of undetermined and/or undescribed *Halotydeus* spp. are known for this region); *Tyrophagus putrescentiae* (Schrank, 1781) (2/5). However, to date, these species have not been reported to contain *Wolbachia* using more reliable methods.

## DISCUSSION

In light of the lack of specific reports of *Wolbachia* in our literature search, or of sequence assignments in the databases examined, we now consider evidence for the wider presence of *Wolbachia* infection in each of the main higher taxonomic groups of terrestrial invertebrates present in Antarctica. We also consider hypotheses relating to the apparent absence of *Wolbachia* in Antarctica.

### Acari

More than a hundred species of Acari (including both Parasitiformes and Acariformes) are recorded from Antarctica (Pugh, 1993; RAS, 2024; Russell et al., 2014). We consider only the non‐parasitic terrestrial mites here. Acariformes include the Sarcoptiformes (Oribatida, Astigmata, Endeostigmata) and several families of Trombidiformes, while free‐living Parasitiformes are represented only by the Mesostigmata. To date, no data on the presence of *Wolbachia* in representatives of these groups are available from samples collected in the Antarctic region.

Most Oribatida are saprophagous or fungivorous. Oribatid mites occur globally (Subías et al., 2012), with more than 80 species recorded from Antarctica (Pugh, 1993). In non‐Antarctic species, *Wolbachia* strains from supergroup E have been reported in seven species of oribatid mite sampled in Poland and the USA (*Oppiella nova* Oudemans, 1902, *Gustavia microcephala* Nicolet, 1855, *Ceratozetes thienemanni*, *Damaeus onustus* Koch, 1844, *Hypochthonius rufulus* Koch, 1835, *Achipteria coleoptrata* Linnaeus, 1758 and *Microzetorchestes emeryi* Coggi, 1898) (Konecka, 2022; Konecka & Olszanowski, 2019). The relationships between *Wolbachia* and their known oribatid hosts have not been studied. However, *Wolbachia* infections were found in both parthenogenetic and sexually reproducing species (Konecka, 2022), possibly suggesting their function in these hosts does not relate to manipulating reproduction.

A few free‐living Astigmata are present in Antarctica although, other than limited occurrence records, these have not been the subject of any specific study, including of the presence of *Wolbachia*. Representatives of the genera *Acarus* Linnaeus, 1758 and *Tyrophagus* Oudemans, 1924 in Antarctica are of particular interest because of the finding of *Wolbachia* (possibly supergroup I) in the globally widespread species *Acarus siro* Linnaeus, 1758 (Hubert et al., 2021) and *Tyrophagus putrescentiae* (Schrank, 1781) (Erban et al., 2016). However, we did not find the bacteria in five samples of *T. putrescentiae* included in our search. According to Erban et al. (2016), the presence of *Wolbachia* is influenced by diet and habitat. Both these mite species have been recorded from the Antarctic region, although no recent taxonomic or molecular studies are available to confirm their identification (RAS, 2024).

The Sarcoptiformes are represented in Antarctica by the families Nanorchestidae and Pachygnathidae (RAS, 2024). The presence of *Wolbachia* in representatives of either family, globally or in Antarctica, has not been reported. However, *Wolbachia* has been reported in the gall‐inducing species *Fragariocoptes setiger* Nalepa, 1894 (Eriophyoidea), which some authors consider to be included in this group (Klimov et al., 2022).

Non‐parasitic terrestrial Trombidiformes present in Antarctica include multiple species representing several families (Pugh, 1993). We only consider taxa here for which *Wolbachia* infection has been confirmed elsewhere. The family Cheyletidae includes the cosmopolitan parthenogenetic species *Cheyletus eruditus* Schrank, 1781, which is also recorded from the Antarctic region (although, given its biology, it is likely to be a synanthropic species). These predatory mites are often found in stored products and are used in biological control measures against grain mites. Hubert et al. (2016) reported the presence of *Wolbachia* in *C. eruditis*.

In the family Tetranychidae (spider mites), infection by *Wolbachia* is widely reported, causing CI (generally supergroup B, with only one mite species infected by a member of supergroup K) (Gotoh et al., 2003; Ros et al., 2009). Notably, the strength of CI is related to annual temperature (Zhu et al., 2021). Weeks and Breeuwer (2001) reported a unique pattern of *Wolbachia* infection associated with parthenogenesis in six species of the phytophagous mite genus *Bryobia* Koch, 1836, namely that parthenogenetic females produced more males in their offspring after tetracycline treatment. However, *B. praetiosa* Koch, 1835 is the only member of the Tetranychidae reported in the Antarctic region (Pugh, 1993). Little is known about its biology in Antarctica, and no studies have addressed the presence/absence of *Wolbachia* in Antarctic material of this species.

Globally, the free‐living Mesostigmata are also characterized by high species diversity and diverse ecological preferences, although few species are present in Antarctica (Pugh, 1993). However, virtually no information is available on the presence of *Wolbachia* in free‐living Mesostigmata, other than that the presence of supergroup B has been noted in Phytoseiidae (Enigl & Schausberger, 2007).

### Collembola

Collembola (springtails) are one of the main components of the soil biota, particularly in the polar regions (Convey & Biersma, 2024), and also one of the most diverse groups of Hexapoda. Due to their significant contribution to soil ecosystem functioning, springtails have been extensively studied.

Research indicates that Collembola emerged approximately 180 million years ago when the global climate was much warmer. Despite being exposed to the harsh conditions which have typified the Antarctic continent since the Miocene, and in particular the repeated ice sheet extent maxima which covered the large majority of Antarctica between the mid‐Miocene and the present, they survived in the remaining ice‐free refugia on the continent (Bartalos, 2018; Convey et al., 2020).

The overall species diversity of Collembola in the Antarctic is low compared to other parts of the globe. Around 25 species are currently recognized to occur on the continent, with this number changing rapidly in recent years with the application of molecular analyses to an increasing number of genera. A strong and ancient biogeographic divide is present at the base of the Antarctic Peninsula, separating the fauna of the maritime Antarctic from that of the continental Antarctic at species level (Chown & Convey, 2007; Convey et al., 2020). Continental Antarctic species are now realized to be endemic to the continent, and in most cases to much smaller specific regions within the continent, while a number of maritime Antarctic species are also regionally endemic. The previously held view that one Collembola species was shared between the maritime and continental Antarctic, *Friesea grisea* Schaffer, 1891 (Neanuridae), is now accepted to be incorrect, with *F. grisea* now restricted to its type location of sub‐Antarctic South Georgia, all maritime Antarctic material previously referred to as *F. grisea* now assigned to *F. antarctica* (Greenslade, 2018), and several new species being erected for representatives of *Friesea* from different regions within continental Antarctica (Carapelli, Cucini, et al., 2020a; Carapelli, Greenslade, et al., 2020b; Stevens et al., 2021). Recent molecular studies of both Collembola and Acari in Victoria Land indicate that further unrecognized and likely species‐level diversity is yet to be described in this region (Brunetti, Siepel, Convey, et al., 2021a; Brunetti, Siepel, Fanciulli, et al., 2021b; Collins et al., 2019, 2020, 2023), and this is similarly likely to be the case for the currently widespread nominate species *Cryptopygus antarcticus* V.Willem, 1901 in the maritime and sub‐Antarctic, where multiple sub‐species are currently recognized based on morphological taxonomy.

There is a greater diversity of Collembola in the milder but still chronically cool sub‐Antarctic islands. The South Georgian Collembola fauna includes at least 23 species (Convey et al., 1999; Greenslade & Convey, 2012), with 35 species on Macquarie Island (Greenslade & Convey, 2012), 28 on Crozet Island (Deharveng, 1981; Greenslade & Convey, 2012), 31 on the Kerguelen archipelago (Deharveng, 1981; Greenslade & Convey, 2012), 16 on Marion Island (Gabriel et al., 2001; Greenslade & Convey, 2012) and eight species on Heard Island (Greenslade & Wise, 1984). Furthermore, a number of non‐native Collembola are known to have been introduced to and become established in the sub‐Antarctic region (Greenslade & Convey, 2012; Phillips et al., 2017). In the maritime Antarctic, *Protapharura* sp., *Folsomia candida* V.Willem, 1902 and *Mucrosomia caeca* (Wahlgren, 1906) (formerly known as *Cryptopygus caecus* Wahlgren, 1906; Potapov et al., 2020), species characteristic of regions with a temperate climate, were reported from geothermally heated ground on Deception Island (South Shetland Islands) in the 1960s, but recent reports of the former two species are not available (Downie et al., 2000; Greenslade, 1995; Hughes et al., 2015). The latter species is thought to be native to such geothermal areas, also being abundant around similar features in the South Sandwich Islands (Convey et al., 2000). The cosmopolitan invasive species, *Hypogastrura viatica* Tullberg, 1872, is now common on Deception Island; it was also formerly reported from Léonie Island (Greenslade, 1995) but was not observed in more recent detailed surveys of the island (Hughes et al., 2017).

Although the species diversity of Collembola in the Antarctic is low compared to other parts of the globe, in the terrestrial habitats in which they occur they can reach densities of tens to hundreds of thousands of individuals per square meter. As with the micro‐invertebrate groups and the Acari, with the recent molecular phylogenetic advances noted above, it is now clear that high levels of species endemism, often at small geographical scales within the continent, characterize the Antarctic collembolan fauna. As noted by Greenslade (2015), the majority of Antarctic Collembola represent the Isotomidae, a family whose representatives are widespread globally (Frati & Carapelli, 1999).

Based on the successful establishment of multiple non‐native Collembola on islands in the sub‐Antarctic region, Greenslade and Convey (2012) produced one of the first risk assessments for invasion by members of this group further south into the maritime Antarctic. Two of the highest risk species identified in that assessment are now already known to be established on the geothermally warmed Deception Island in the South Shetland Islands, along with four other non‐native Collembola (Greenslade et al., 2012; Hughes et al., 2015).

To date, *Wolbachia* infection (supergroup E) has been reported globally mainly in parthenogenetic collembolan species, including members of the Tullbergiidae (Poduromorpha) (*Mesaphorura italica* Rusek, 1971, *M. macrochaeta* Rusek, 1976 and *Paratullbergia callipygos* Börner, 1902), Isotomidae (Entomobryomorpha) (*F. candida* and *Parisotoma notabilis* Schäffer, 1896) and Neelidae (Neelipleona) (*Megalothorax minimus* V.Willem, 1900 and *Neelus murinus* J.W.Folsom, 1896) (Czarnetzki & Tebbe, 2004; Ma et al., 2017; Tanganelli et al., 2014; Timmermans et al., 2004; Vandekerckhove et al., 1999). Of these, *M. macrochaeta* and *F. candida* are among the non‐native species that have been recorded from Antarctica (Greenslade et al., 2012; Hughes et al., 2015). The sexually reproducing species, *Orchesella cincta* C.Linnaeus, 1758 (Entomobryomorpha, Entomobryidae) and *Anurida maritima* Guérin‐Méneville, 1836, also display *Wolbachia* infection (Ma et al., 2017; Timmermans et al., 2004, 2023). The latter springtail species consists of divergent lineages and some authors consider it to be a species group (Sun et al., 2018). Analyses of *cif* genes from *Wolbachia* genomes from different springtail lineages revealed no involvement of CI in genetic divergence within the species group (Timmermans et al., 2023).

The divergent *Wolbachia* supergroup E (Vandekerckhove et al., 1999) is predominantly found in asexually reproducing Collembola (springtails), where it has been associated specifically with parthenogenesis induction (Ma et al., 2017). Pike and Kingcombe (2009) and Timmermans and Ellers (2009) reported that complete loss of *Wolbachia* infection in *F. candida* led to the production of normal clutch sizes but with complete failure of egg hatching, demonstrating that *F. candida* is strictly dependent on *Wolbachia* presence to produce viable offspring.

Among the collembolan species identified in the literature search from which *Wolbachia* has been reported, four species are now present and considered non‐native in the Antarctic region: *M. macrochaeta* (found on Deception Island [Greenslade et al., 2012] and Macquarie Island [Greenslade & Convey, 2012]), *F. candida* (Deception Island [Greenslade, 1995; Greenslade & Convey, 2012]), *P. notabilis* (Marion Island [Greenslade & Convey, 2012]) and *M. minimus* (Kerguelen archipelago and Marion Island [Greenslade & Convey, 2012]) (Timmermans et al., 2023). However, no Antarctic material of these species has been examined for the presence of *Wolbachia*. No evidence of *Wolbachia* presence was found in SRA libraries generated from one sample of *Desoria tigrina* Nicolet, 1842 and 18 samples of *F. candida* (Table S2). However, six and seven of the studied *F. candida* libraries contained *Wolbachia* according to the NCBI SRA classifier and Kraken2, respectively (all samples were collected outside the Antarctic region) contained *Wolbachia*. Such a difference may indicate that the mOTU3 profiler may not be sensitive enough for detection and its results cannot be considered conclusive.

### Diptera

The chironomid midges *Belgica antarctica* Jacobs, 1900 and *Parochlus steinenii* Gercke, 1889 (Podonominae) are the only insect species native to the Antarctic continent, although a larger diversity of native insects is present on the sub‐Antarctic islands (Allegrucci et al., 2006; Chown & Convey, 2016; Convey & Block, 1996). Two further non‐native insects are established in parts of the maritime Antarctic, the chironomid *Eretmoptera murphyi* Schäffer, 1914 (Orthocladiinae; a species endemic to sub‐Antarctic South Georgia) on Signy Island (South Orkney Islands) and *Trichocera maculipennis* Meigen, 1818 (Trichoceridae) on King George Island (South Shetland Islands) (Chown & Convey, 2016; Potocka & Krzemińska, 2018; Remedios‐de León et al., 2021; Volonterio et al., 2013). Diptera in general are commonly infected with *Wolbachia* (supergroups A and B), where the endosymbiont can manipulate host reproduction through CI, MK and parthenogenesis induction (O'Neill et al., 1997; Werren et al., 2008). *Wolbachia* can also provide a broad range of protection against pathogens including viruses, bacteria, filarial nematodes and the malarial parasite *Plasmodium* (Kaur et al., 2021), as well as having a wide range of other effects (Deconninck et al., 2024; Saeed et al., 2018; Weeks et al., 2007; reviewed in Serga et al., 2023).

Notwithstanding the widespread occurrence of *Wolbachia* in Diptera, there is only a single report in the Chironomidae, from an unidentified species from Naperville (USA) (NeuquaValley, 2021). Our own analyses of 44 specimens of *B. antarctica* from several locations in the Antarctic Peninsula region via PCR and whole genome data from 34 samples gave no evidence of the presence of *Wolbachia* (Maistrenko et al., 2023), as also reported by Holmes et al. (2019). Of note, *B. antarctica* is a sexually reproducing species, while the closely related *E. murphyi* (which molecular phylogenetic analyses place as sister species within a clade that otherwise only contains the two described species in the genus *Belgica* [Allegrucci et al., 2012]) is obligately parthenogenetic. The two species are palaeoendemic on distinct tectonic elements (Antarctic Peninsula and South Georgia, respectively), with divergence possibly coincident with the final stages of breakup of the supercontinent Gondwana over 30 million years ago. Whether *Wolbachia* has played a role in the adoption of parthenogenesis in *E. murphyi* has never been studied. Similarly, the four whole genome sequences of *P. steinenii* included in our analyses gave no evidence of *Wolbachia* presence (Table S2). No cases of *Wolbachia* infection have been reported in the literature in the taxonomic unit Trichoceroidea, reflecting a lack of research to date.

The currently limited available data suggest that *Wolbachi*a are not prevalent in Chironomidae (among 10 tested samples of Chironomidae in Weinert et al.'s (2015) database, one was infected). However, our bioinformatics search provided weak evidence (only one sample with total coverage <0.01% of its genome of *Wolbachia*; Table S2) suggesting that *Wolbachia* might be present in one sample of *B. antarctica*. It is important to note that direct PCR testing did not confirm *Wolbachia* infection in this species. Thus, there is a need for more in‐depth research using comprehensive, evidence‐based methods to search for this endosymbiont in Antarctic Diptera.

### Rotifera

Rotifers (Phylum Rotifera, also referred to as Syndermata) are pseudocoelomate unsegmented invertebrates widely distributed across virtually all regions and habitats on Earth. They are one of the key groups of Antarctic microfauna in lakes, wetlands and terrestrial habitats. Rotifera are microscopic (smaller than 2 mm) and comprise three large taxonomic groups (Segers, 2002). Seisonacea (Subclass Pararotatoria), which are epizoic on marine crustaceans, Monogononta and Bdelloidea (Subclass Eurotatoria), which are mostly free‐living although include several endoparasitic species in invertebrate hosts. Recently, it has been confirmed that thorny‐headed worms, macroscopic obligate endoparasites formerly referred to the Phylum Acanthocephala, are modified rotifers, and they are now included within the Seisonacea (Sielaff et al., 2016; see also Ricci, 1998). Seisonacea currently comprises only two genera each containing two species. The most species‐rich group of rotifers is Monogononta, which includes ~1760 described species and subspecies belonging to 115 genera, followed by Bdelloidea, with 20 genera and more than 460 clonal species (including former ‘subspecies’). However, it should be noted that modern taxonomic knowledge of these groups is limited, and many currently undescribed species are likely to exist, particularly in poorly researched regions such as Antarctica (Iakovenko et al., 2015).

Monogonont rotifers are present in water bodies of all types (including brackish and marine), with a relatively small number of limno‐terrestrial species. Seisonacea (Subclass Pararotatoria) are epizoic on marine crustaceans, and do not occur in Antarctica. Monogononta are mostly free‐living except for a few endoparasitic species in invertebrate hosts; in Antarctica they are associated with lakes and other water bodies. All Bdelloidea are free‐living and occur both in aquatic and terrestrial Antarctic habitats, with prevalence in the latter. They show well developed stress adaptations, although those occurring in freshwater habitats generally experience a relatively more stable environment during the polar summer months than those present in terrestrial habitats. They enter diapause at the stage of fertilized eggs (heavily encapsulated embryos or resting/diapausing eggs) when habitat conditions become unfavourable. Population density and photoperiod are among the drivers of diapause entry (Pourriot & Clément, 1981). The life cycle of monogononts is heterogonic, with generations of female parthenogenesis and bisexual reproduction, or mixis (Serra & Snell, 2009). Parthenogenetic reproduction includes many cycles of production of diploid eggs, while mixis results in production of haploid eggs, haploid males and, after fertilization, the restoration of diploidy in diapausing eggs which then undergo a long dormant period (Gilbert, 2020). Monogonont males are small, underdeveloped (commonly with reduction in all systems except those relating to reproduction) and live for only a few days; their only function is fertilizing the female. Unlike monogononts, bdelloid rotifers are commonly found in terrestrial habitats (e.g., moss, lichen, soil, forest litter, decomposing wood, macroscopic fungi) which can frequently (up to several times a day) experience wet‐dry cycles and freeze–thaw cycles in polar ecosystems. These very short periods of activity are too narrow to enable production of diapausing eggs, while parthenogenetic bdelloid eggs, although often able to tolerate desiccation (Örstan, 1995), have relatively low resistance to adverse conditions in comparison with the resting eggs of monogononts. Consequently, bdelloids withstand desiccation and freezing as adults, with well‐developed anhydrobiotic abilities. All bdelloid rotifers are believed to be obligately parthenogenetic, with males never described (although this does not exclude meiosis in females). Finally, seisonacean rotifers are dioecious with obligate bisexual reproduction; male and female Seisonacea are equally developed and occur in approximately equal proportions on their symbiotic or semi‐parasitic host (Ricci et al., 1993).

Both Bdelloidea and Monogononta have been known from the Antarctic region since the reports of the earliest Antarctic expeditions (David et al., 1910; Richters, 1904). Since then, more than 90 publications have documented at least 154 species of rotifer present in Antarctica (Fontaneto et al., 2015), with 12 new species recently described (Iakovenko et al., 2015). A brief history of Antarctic rotifer research, biodiversity surveys, and details on biology can be found in Dartnall and Hollowday (1985) and more recent publications (Adams et al., 2006; Convey et al., 2008; Convey & Stevens, 2007; Dartnall, 2017; Fontaneto et al., 2015; Iakovenko et al., 2015; Lukashanets et al., 2021; Velasco‐Castrillón et al., 2018; Velasco‐Castrillón, Page, et al., 2014b). Traditional taxonomic approaches based on morphological analyses have often led to the miss‐naming of bdelloids present in Antarctica, using the names of taxa originally described from Europe. For instance, *Adineta editae* Iakovenko, 2015—a bdelloid common in Antarctic soil and moss—was long referred to in faunistic reports as *A. gracilis* Janson, 1893 (described from a sphagnum swamp in Germany), until Iakovenko et al. (2015) demonstrated that it is a morphologically and genetically distinct species. Velasco‐Castrillón, Page, et al. (2014b) and Iakovenko et al. (2015), using mitochondrial *cox1* gene analysis, have demonstrated unexpectedly high levels of biodiversity and endemism in Antarctic rotifers in comparison with those from any other continent.

No reports of the presence of *Wolbachia* in Rotifera were found in our literature search. We also did not find any evidence supporting *Wolbachia* presence in bacterial profiles of SRA libraries obtained from *A. steineri* Bartoš, 1951 (*n* = 4), *A. vaga* (Davis, 1873) (*n* = 28), *B. calyciflorus* Pallas, 1766 (*n* = 10), *B. angularis* Gosse, 1851 (*n* = 2) and *Macrotrachela quadricornifera* Milne, 1886 (*n* = 4) (Table S2). Given the considerable emphasis on parthenogenetic reproduction in Rotifera, the hypothesis that *Wolbachia* or other reproductive parasites might underlie this feature cannot be ruled out.

### Nematoda

Nematodes are one of the richest and most abundant groups of terrestrial fauna in Antarctica. Around sixty terrestrial nematode species are currently reported to occur in Antarctica, representing 16 families and almost 30 genera (Andrássy, 1998; Elshishka et al., 2023; Maslen & Convey, 2006; Raymond et al., 2014). Further genera have been reported but not identified to the species level, and significant currently undescribed diversity is likely to be present, especially given the very limited effort to date to apply molecular biological approaches in studies of Antarctic nematodes (Kagoshima et al., 2019; Maslen & Convey, 2006). All Antarctic terrestrial nematodes are thought to be free‐living, with no confirmation of the presence of plant‐parasitic species.

The nematode fauna of Antarctica is clearly distinct from that of other continents. At least 90% of species currently known, and possibly approaching 100%, are endemic to the continent or smaller regions within it, indicating a long evolutionary history in isolation within the continent, as is now known to be characteristic of most Antarctic terrestrial fauna (Andrássy, 1998, 2008; Convey et al., 2020; Maslen & Convey, 2006). For example, all six species representing the large genus *Eudorylaimus* Andrássy, 1959 (Qudsianematidae) in the nematode fauna of continental Antarctica (with around 100 species known globally) are endemic (Andrássy, 2008). Only a very few members of the continent's nematode fauna are currently referred to cosmopolitan species, including *Eumonhystera vulgaris* (de Man, 1880) Andrássy, 1981, *Geomonhystera villosa* (Bütschli, 1873) Andrássy, 1981 and *Ceratoplectus armatus*, (Bütschli, 1873) Andrássy, 1981. However, none of these has been subjected to molecular analyses. If confirmed, these species may have a non‐Antarctic origin with introduction through human assistance (Andrássy, 1998).

Intra‐Antarctic regional endemism is strongly apparent in the continent's nematode fauna, with the maritime and continental Antarctic fauna being almost completely distinct, as noted above a feature shared across all Antarctic terrestrial faunal groups (Chown & Convey, 2007; Convey et al., 2020; Maslen & Convey, 2006; Short et al., 2022). The nematode fauna of the maritime Antarctic primarily comprises the families Teratocephalidae, Rhabditidae, Aphelenchoididae, Alaimidae, Mononchidae, Dorylaimidae and Nordiidae, while the Cephalobidae, Panagrolaimidae and Tobrilidae dominate the fauna of continental Antarctica. Three families are present in both regions, the Monhysteridae, Plectidae and Qudsianematidae, but no individual species have been confirmed to be present in both regions (Andrássy, 1998; Elshishka et al., 2015; Kito & Ohyama, 2008; Maslen & Convey, 2006).

A recent study of the gut microbiota of two Antarctic nematodes (*Plectus murrayi* Yeates, 1970 and *E. antarcticus* (Steiner, 1916) Yeates, 1970) did not detect the presence of *Wolbachia* in their microbiomes (McQueen et al., 2022). However, bacteria within the sister family of Rickettsiaceae were detected at very low abundance (McQueen et al., 2022). In our own analyses *Wolbachia* was absent from *Anisakis simplex* (Rudolphi, 1809) Dujardin, 1845 (synonym of *A. pegreffii* Campana‐Rouget & Biocca, 1955; *n* = 3), *Aphelenchus avenae* Bastian, 1865 (*n* = 3) and *P. murrayi* (*n* = 1) (Table S2).

Elsewhere globally, *Wolbachia* occurs widely in parasitic filarial nematodes (Manoj et al., 2021) and has also been reported in plant parasitic nematodes (Brown et al., 2018; Haegeman et al., 2009; Weyandt et al., 2022), but it has not been found in free‐living nematodes (Bordenstein et al., 2003). In plant parasitic nematodes, *Wolbachia* were first identified in *Radopholus similis* (Cobb, 1893) Thorne, 1949, but their effects on the host remain unknown (Haegeman et al., 2009). *Wolbachia* is a facultative symbiont of *Pratylenchus penetrans* (Cobb, 1917) Filipjev & Schuurmans Stekhoven, 1941, leading to female‐skewed sex ratios in infected populations (Wasala et al., 2019). *Wolbachia* has also recently been identified in plant parasitic nematode communities, including in *Helicotylenchus* spp. and *Rotylenchus* spp. (Weyandt et al., 2022). Strains of *Wolbachia* from these hosts belong to the L supergroup while those from filarial nematodes are members of the C, D and F supergroups (Haegeman et al., 2009). Screening of the SRA database with 3400 available samples from soils and rhizospheres confirmed the presence of *Wolbachia* reads in 24 SRA runs from different sampling locations globally, but none from Antarctica. Nineteen of these clustered with previously described *Wolbachia* from plant‐parasitic nematodes (Weyandt et al., 2022). Overall, there is a limited number of studies available that have targeted *Wolbachia* presence in nematodes.

### Tardigrada

Tardigrada Doyère, 1840 (commonly known as water bears), which includes the classes Eutardigrada and Heterotardigrada, are microscopic invertebrates closely related to Arthropoda and Onychophora (Giribet & Edgecombe, 2012). They are present in almost all freshwater, marine and terrestrial habitats throughout the world. They are commonly found in association with mosses, lichens, hepatics, algae, soil, litter, freshwater and marine sediments and aquatic plants, and occasionally also on higher terrestrial plants (Nelson et al., 2015). More than 1400 tardigrade species and sub‐species are presently described (Degma & Guidetti, 2009–2023).

Since the beginning of the 20th century, more than 70 eutardigrade and heterotardigrade species have been reported from Antarctica. Some, possibly many, of these species are thought to be endemic to Antarctica, while others have been suggested to be cosmopolitan species or even accidentally introduced by humans, however, as with other Antarctic micro‐invertebrate groups, most Antarctic tardigrades have yet to be subjected to molecular phylogenetic analyses (Binda et al., 2005; Convey & McInnes, 2005; Dastych, 1984, 2018; Guidetti et al., 2014, 2017; Kaczmarek et al., 2014, 2018; Kaczmarek, Mioduchowska, et al., 2020a; Kihm et al., 2020; Lukashanets et al., 2021; McInnes, 2010; Mioduchowska, Kačarević, et al., 2021a; Nelson et al., 2020; Pilato et al., 2012, 2017; Robertson et al., 2020; Short et al., 2022; Tsujimoto et al., 2014, 2020; Tumanov, 2022; Vecchi, Cesari, et al., 2016a; Velasco‐Castrillón, Gibson, & Stevens, 2014a).

A number of recent studies of tardigrade microbiomes are now available, including some of Antarctic species (Guidetti et al., 2020; Kaczmarek, Roszkowska, et al., 2020b; McQueen et al., 2022, 2023; Mioduchowska, Nitkiewicz, et al., 2021b; Tibbs‐Cortes et al., 2022; Vecchi et al., 2018; Vecchi, Vicente, et al., 2016b). However, reports of the presence of *Wolbachia* in tardigrades remain very limited and mostly opportunistic. Mioduchowska, Nitkiewicz, et al. (2021b) reported *Wolbachia* infection in adults of two species of tardigrades, with their most recent study confirming these belonging to the A supergroup (Mioduchowska et al., 2022, 2023). At present, *Wolbachia* infection has been confirmed in only four eutardigrade taxa, *Paramacrobiotus* sp. (from Poland) *P. experimentalis* Kaczmarek, Mioduchowska, Poprawa and Roszkowska, 2020 (from Madagascar), *Macorobiotus basiatus* Nelson, Adkins Fletcher, Guidetti, Roszkowska, Grobys and Kaczmarek, 2021 (from USA) and *M. polypiformis* Roszkowska, Ostrowska, Stec, Janko and Kaczmarek, 2017 (from Ecuador) (Mioduchowska, Nitkiewicz, et al., 2021b; Mioduchowska et al., 2022, 2023), all members of the superfamily Macrobiotoidea. There are no confirmed occurrence records of *M. polypiformis* in the Antarctic region, but at least 10 other representatives of this genus have been recorded in Antarctica (Velasco‐Castrillón, Gibson, & Stevens, 2014a). Representatives of *Paramacrobiotus* are also present in Antarctica, such as *P. fairbanksi* Schill, Förster, Dandekar and Wolf, 2010; nevertheless, *Wolbachia* was not found in non‐Antarctic material this species (Mioduchowska, Nitkiewicz, et al., 2021b). *Wolbachia* has also been reported in the microbiome of undetermined tardigrades from the USA (Tibbs‐Cortes et al., 2022). However, *Wolbachia* infection was not detected in the gut microbiomes of 94 undetermined tardigrade specimens (likely to represent only a single species; collected from cyanobacterial mats from streams in Taylor Valley, Antarctica) using 16S and 18S rRNA metabarcoding (McQueen et al., 2022). Members of the bacterial order Rickettsiales have also been reported in association with adults and/or eggs in tardigrades collected in Antarctica (McQueen et al., 2022; Mioduchowska, Nitkiewicz, et al., 2021b), including *P. fairbanksi* (Mioduchowska, Nitkiewicz, et al., 2021b) and *Acutuncus antarcticus* Richters, 1904 (Vecchi et al., 2018). Similarly, association with Rickettsiales has been reported for the European species *Echiniscus trisetosus* Cuénot, 1932, *Ramazzottius oberhaeuseri* Doyère, 1840, *Richtersius coronifer* Richters, 1903, *Macrobiotus macrocalix* Bertolani and Rebecchi, 1993 and *Paramacrobiotus areolatus* Murray, 1907 (Vecchi et al., 2018). These species are also currently recorded in Antarctica, although they have not been confirmed by modern molecular phylogenetic analyses. Finally, we profiled 10 available SRA libraries from laboratory cultured *Hypsibius dujardini* Doyère, 1840 and did not detect the presence of *Wolbachia* (Table S2).

Unlike marine tardigrades, for most of which parthenogenesis is unknown, this form of reproduction is common in limno‐terrestrial tardigrades (Bertolani, 2001), including in the Antarctic species *A. antarcticus* (Altiero et al., 2015). In some tardigrade taxa (e.g., Family Murrayidae), males are unknown, while in others (e.g., genus *Echiniscus* C.A.S. Schultze, 1840), males are frequent only in Australia and Antarctica, are rare in Asia and absent on other continents (Bertolani, 2001). As with other groups considered here in which parthenogenesis is an important reproductive feature, it is not yet known whether cases of parthenogenesis in tardigrades are related to *Wolbachia* infection.

### *Hypotheses for the apparent absence of* Wolbachia *in Antarctica*

Our literature searches and analyses provided no direct evidence that *Wolbachia* is present in Antarctic terrestrial invertebrates. Moreover, we have found no studies mentioning *Wolbachia* presence in eDNA data obtained from Antarctic soil samples, while the PubMed search of Rickettsiales order in Antarctica gave no relevant results.

Similarly, our analysis indicates that no *Wolbachia* have been detected to date in the Arctic. However, pathogens representing the order Rickettsiales have recently been reported in tick samples from regions close to the Arctic Circle. For instance, *Anaplasma phagocytophilum* (Henningsson et al., 2015), *Candidatus Neoehrlichia mikurensis* (Jenkins et al., 2019) and *Rickettsia helvetica* (Hvidsten et al., 2020) were reported in tick specimens from the Norwegian Arctic, while *Ehrlichia khabarensis* was detected in Alaska (Hahn et al., 2023).

**FIGURE 2:**
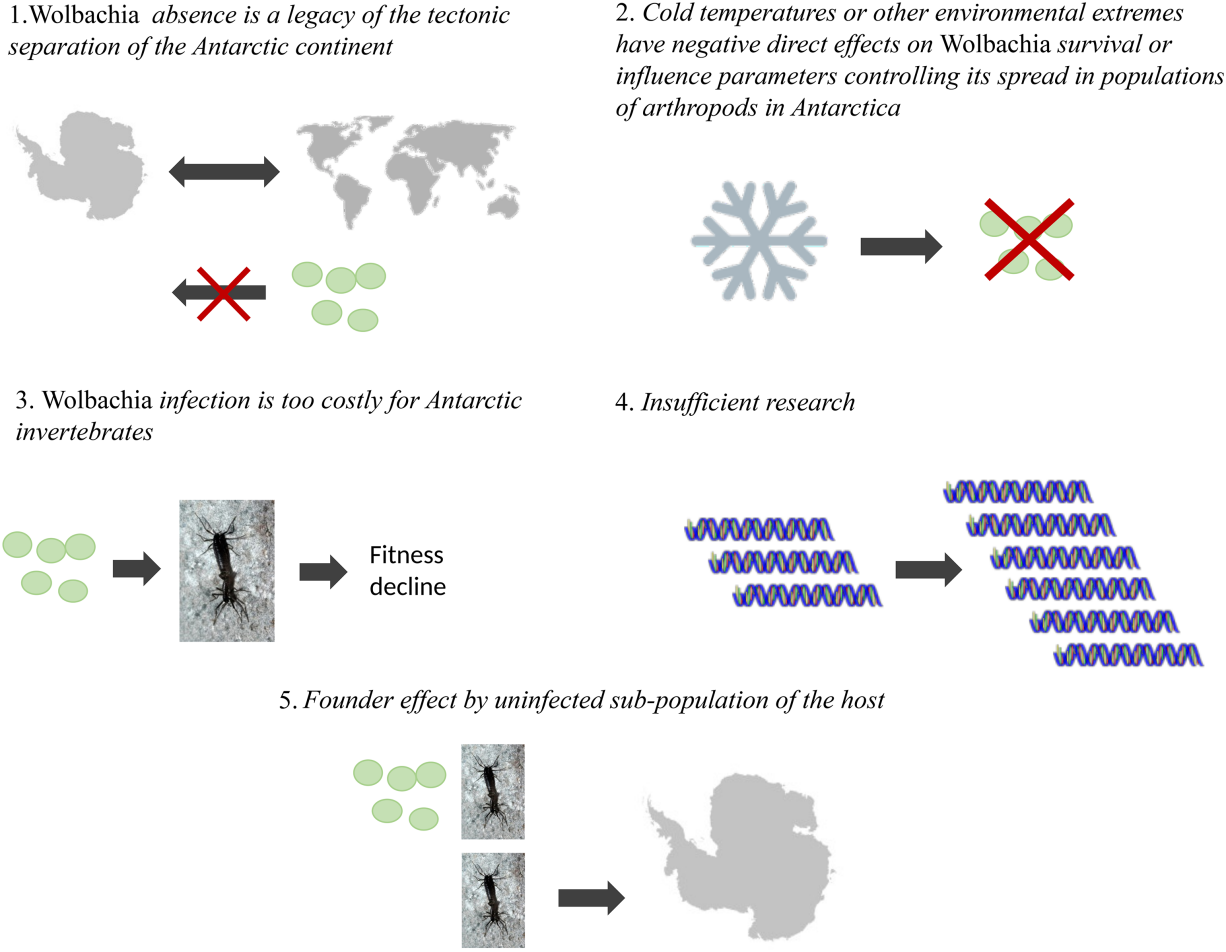
Hypotheses about the absence of *Wolbachia* in invertebrates in Antarctic biomes. Endosymbiotic bacteria *Wolbachia* are recognized for their role in influencing host survival and stress resistance, particularly in cold environmental conditions across various species. However, our analysis of available data reveals a notable absence of Wolbachia in species abundant in the extreme cold conditions of Antarctica.

Below, we consider five possible hypotheses for this finding (Figure 2) and highlight future avenues of research that could explicitly resolve this ongoing debate.

#### *Wolbachia* absence is a legacy of the tectonic separation of the Antarctic continent

Divergence time analyses indicate that *Wolbachia* diverged from two outgroup genera (*Anaplasma* Theiler, 1910 and *Ehrlichia* Moshkovski, 1945) ~1799 million years ago (Mya), while the arthropod‐infecting supergroups A and B are reciprocally monophyletic and diverged from their most recent common ancestor 217 Mya (Liu et al., 2023). While there are no explicit data for the entire subsequent period on *Wolbachia* occurrence or prevalence on the Antarctic continent it seems very unlikely, given patterns of global climate and biological distribution over much of that period, that *Wolbachia* has never been present on what became Antarctica. Rather, as all but the last remaining vestiges of Antarctica's original biodiversity were driven extinct once the continent started the cooling that led to the formation of its vast ice sheets (Convey et al., 2018), *Wolbachia* was similarly lost from the continent, either through being unable to tolerate the changing conditions itself, or by the loss of its then host organisms.

#### Phylogeography and demographic events in the host populations negatively influence *Wolbachia* spread and maintenance in Antarctica

Phylogeography and population structure of host species have significant effects on *Wolbachia* spread and abundance. *Wolbachia* should spread more rapidly in host species that disperse over long distances compared to those that disperse over shorter distances (Turelli & Hoffmann, 1995). Most terrestrial invertebrates inhabiting Antarctica are endemic or non‐cosmopolitan species with limited sharing of nematodes, mites and collembolan species between the continent and Antarctic Peninsula, which prevents infection between the populations (Convey et al., 2020; Greenslade, 2018; Maslen & Convey, 2006; Pugh & Convey, 2008). It has been predicted that *Wolbachia* infection is expected to spread more slowly in subdivided host populations compared to panmictic populations (Wade & Stevens, 1994). Available data from mtDNA or whole‐genome sequencing analyses suggest that populations of Antarctic midges, springtails and mites are highly regionally structured (Allegrucci et al., 2012; McGaughran et al., 2010, 2019; van Vuuren et al., 2018). Nevertheless, migration events can occur between island populations (Edgington et al., 2023).

Demographic events themselves could lead to the loss of *Wolbachia* infection. Colonization or invasion of new habitats could be accompanied by decrease of *Wolbachia* abundance in populations of different insect species (Nguyen et al., 2016; Shoemaker et al., 2000; Tsutsui et al., 2003). Mechanisms behind this decrease of infection frequencies or even loss of infection are not well understood. Founder effects or genetic drift provide potential explanations, when a few individuals establish a new population. Alternatively, it could be due to the selective disadvantages faced by *Wolbachia*‐infected individuals (Turelli & Hoffmann, 1995). However, founder and drift effects could have the opposite influence, increasing stochastically the frequency of *Wolbachia* in the population (Turelli & Barton, 2022; Turelli & Hoffmann, 1995). Founder events can have more significant influences on *Wolbachia* abundance in host species that undergo repeated population extinction and recolonization (Turelli & Hoffmann, 1995). More information is required to reconstruct founder effects in populations of Antarctic invertebrates, particularly using whole‐genome sequencing data.

#### Cold temperatures or other environmental extremes have negative direct effects on *Wolbachia* survival or influence parameters controlling its spread in populations of arthropods in Antarctica

*Wolbachia* titers are highly variable and temperature‐sensitive (Chrostek et al., 2021; Clancy & Hoffmann, 1998; Foo et al., 2019; Hague et al., 2020). Temperature can also affect *Wolbachia* cells directly. For example, a temperature of 30°C is known to kill *Wolbachia* in isopods (Rigaud & Juchault, 1998). However, how low temperature affects *Wolbachia* survival is currently unknown. *Wolbachia* infection has been reported in populations sampled at non‐Antarctic locations with average annual temperatures of 0–6°C (Jaenike et al., 2010; Ritter et al., 2013; Viljakainen et al., 2008). In the absence of explicit evidence, the possibility that different strains or supergroups of *Wolbachia* can survive under different temperature regimes (e.g., mean, range, variability, extremes) cannot be excluded, and more studies are required to understand the impact of temperature on *Wolbachia*.

Two different mechanisms can be hypothesized to explain *Wolbachia* prevalence—its presence within infected species and its spread to new host species (Turelli et al., 2022). *Wolbachia* dynamics in populations of infected species are assumed to depend on the three main parameters of (i) the rate of maternal transmission (1‐*μ*, where *μ* is the frequency of uninfected ovaries produced by an infected female), (ii) the relative fitness of infected individuals (in the simplest case, relative fecundity, *F*) and (iii) the effects on hatch rate (*H*, or strength of CI in case of CI‐induced bacteria) (Hoffmann & Turelli, 1997). All these parameters are sensitive to temperature. For example, in *D. melanogaster*, mild temperature (20°C) has a negative impact on maternal transmission efficiency in comparison with 28°C (transmission efficiency 58.5%–86.6% against 98.1%–99.5%, respectively) (Hague et al., 2022). Temperature is the most important factor influencing the strength of reproductive manipulations, which promote *Wolbachia* spread in the host population (Hurst et al., 2000, 2001; Reynolds & Hoffmann, 2002; Shropshire et al., 2020). Furthermore, *Wolbachia* can have significant impacts on host fitness and its phenotypic expression depends on environmental conditions (Saeed et al., 2018; Strunov et al., 2022). *Wolbachia* can also reduce host cold resistance (Serga et al., 2021). The effects of temperature are clearly highly variable depending on both host species and *Wolbachia* type and require further investigation.

The mechanism of transfer of *Wolbachia* between species remains unclear. While horizontal transmission has been documented (reviewed in Sanaei et al., 2021), the precise mechanisms involved and factors affecting them are far from understood. The CI‐inducing *Wolbachia* has been proposed to transfer more frequently between host species because of their general higher occurrence frequency (Turelli et al., 2022).

Chronic exposure to low temperatures could lead to the loss of *Wolbachia* infection in Antarctica due to imperfect maternal transmission, negative impacts on the expression of reproductive phenotype or negative effects on the fitness of infected individuals. Low infection levels in natural populations in low temperature environments could also restrict interspecific transfer.

Most studies of *Wolbachia*–host relationships focus on temperature effects (Charlesworth et al., 2019; Chrostek et al., 2021; Kriesner et al., 2016). However, other environmental factors can influence *Wolbachia* dispersal and transmission in host populations through direct or indirect mechanisms in an analogous fashion as described for temperature. For example, it has been shown that humidity in combination with temperature can influence rates of *Wolbachia* infection, but the direction of influence depends on the continent or climatic zone (Gora et al., 2020). Generally, in addition to very low temperatures, the Antarctic climate is arid, with low atmospheric humidity and precipitation, and liquid water is present in the simple soils only during the summer period (Campbell & Claridge, 1987; Convey & Biersma, 2024), likely to impact *Wolbachia* dispersal.

#### *Wolbachia* infection is too costly for Antarctic invertebrates

Environmental conditions in Antarctica are characterized by restricted access to key resources, in particular liquid water, nutrients and thermal energy (Block et al., 2009). The host's ability to balance the costs of *Wolbachia* infection with any benefits on aspects of its fitness may, therefore, become a key factor (Martinez et al., 2015). However, low *Wolbachia* density in the host organism can also have very low to marginal costs (Poinsot & Merçot, 1997). Nutrient availability can also influence the host *Wolbachia* titre (Serbus et al., 2015). The characteristically low nutrient availability in Antarctica (Block et al., 2009) could therefore negatively impact the *Wolbachia* titre should it be able to successfully infect Antarctic arthropods. However, as imperfect *Wolbachia* maternal transmission usually occurs in conditions with low *Wolbachia* titers (Hague et al., 2020), low Antarctic nutrient availability environment could negatively impact efficiency of bacterial maternal transmission and promote loss of infection.

#### Insufficient research

Finally, the lack of evidence of *Wolbachia* presence in Antarctica could simply indicate a lack of appropriate research. The number of published studies that have specifically searched for *Wolbachia* in Antarctica is small (seven articles), while negative results are often not published or commented on. It is plausible that infected invertebrate species transferred into Antarctica by zoochory (inadvertent transfer on/in the bodies of birds and mammals) or with anthropogenic assistance may introduce *Wolbachia* to the continent. However, we analysed 155 SRA libraries available in the NCBI GenBank database (July 2023) for the presence of *Wolbachia* in sequenced invertebrate samples. These represent all currently available whole‐genome sequencing data of invertebrate species whose global distributions include Antarctica (however, these include only five samples obtained in Antarctica itself). Our robust search using the mOTU3 profiler did not generate any evidence of the presence of *Wolbachia*. However, the NCBI classifier detected *Wolbachia* in one sample of the Antarctic endemic midge *B. antarctica*. This discrepancy suggests that *Wolbachia* may indeed be present in this species. New research specifically targeting *Wolbachia* presence in a wide range of Antarctic terrestrial invertebrates, particularly in species or genera known to host the bacteria elsewhere globally, and Antarctic species/groups where parthenogenesis is prevalent, is required before robust conclusions can be drawn on the presence, prevalence and controlling factors of *Wolbachia* infection in Antarctica.

## AUTHOR CONTRIBUTIONS

**Svitlana Serga:** Conceptualization (equal); methodology (equal); investigation (equal); formal analysis (equal); visualization (equal); data curation (equal); writing – original draft (lead); writing – review and editing (equal). **Pavlo A. Kovalenko:** Conceptualization (supporting); investigation (equal); formal analysis (equal); visualization (equal); data curation (equal); writing – original draft (equal); writing – review and editing (equal). **Oleksandr M. Maistrenko:** Conceptualization (supporting); methodology (lead); investigation (lead); formal analysis (lead); visualization (lead); data curation (lead); writing – original draft (equal); writing – review and editing (equal). **Gwenaëlle Deconninck:** Investigation (equal); visualization (equal); writing – review and editing (equal). **Oleksandra Shevchenko:** Investigation (equal); writing – original draft (equal); writing – review and editing (equal). **Nataliia Iakovenko:** Investigation (equal); writing – original draft (equal); writing – review and editing (equal). **Yurii Protsenko:** Investigation (equal); writing – original draft (equal); writing – review and editing (equal). **Andrij Susulovsky:** Investigation (equal); writing – original draft (equal); writing – review and editing (equal). **Łukasz Kaczmarek:** Investigation (equal); writing – original draft (equal); writing – review and editing (equal). **Mariia Pavlovska:** investigation (supporting), writing – review and editing (equal). **Peter Convey:** Conceptualization (supporting); investigation (equal); validation (lead); writing – review and editing (lead). **Iryna Kozeretska:** Conceptualization (lead); methodology (equal); validation (equal); writing – original draft (lead); writing – review and editing (equal); project administration (lead).

## CONFLICT OF INTEREST STATEMENT

The authors declare no conflict of interest.

## Supporting information


**TABLE S1.** A database of *Wolbachia* infection screens in Antarctic arthropod species.

**TABLE S2.** Results of *Wolbachia* sequence identification using mOTU3, the NCBI sequence read archive classifier and Kraken2.

**TABLE S3.** Results of prokaryote sequence identification from SRA libraries using the mOTU3 profiler.

## Data Availability

The data that supports the findings of this study are available in the supplementary material of this article.
